# On the Energy Performance of Iridium Satellite IoT Technology

**DOI:** 10.3390/s21217235

**Published:** 2021-10-30

**Authors:** Carles Gomez, Seyed Mahdi Darroudi, Héctor Naranjo, Josep Paradells

**Affiliations:** 1 Department of Network Engineering, Universitat Politècnica de Catalunya, C/Esteve Terradas, 7, 08860 Castelldefels, Spain; sm.darroudi@entel.upc.edu (S.M.D.); hectorj_naranjo20@hotmail.com (H.N.); josep.paradells@entel.upc.edu (J.P.); 2 Fundació i2cat, C/Gran Capità, 2, 08034 Barcelona, Spain

**Keywords:** satellite, IoT, Iridium, energy, model, LoRaWAN, Sigfox

## Abstract

Most Internet of Things (IoT) communication technologies rely on terrestrial network infrastructure. When such infrastructure is not available or does not provide sufficient coverage, satellite communication offers an alternative IoT connectivity solution. Satellite-enabled IoT devices are typically powered by a limited energy source. However, as of this writing, and to our best knowledge, the energy performance of satellite IoT technology has not been investigated. In this paper, we model and evaluate the energy performance of Iridium satellite technology for IoT devices. Our work is based on real hardware measurements. We provide average current consumption, device lifetime, and energy cost of data delivery results as a function of different parameters. Results show, among others, that an Iridium-enabled IoT device, running on a 2400 mAh battery and sending a 100-byte message every 100 min, may achieve a lifetime of 0.95 years. However, Iridium device energy performance decreases significantly with message rate.

## 1. Introduction

In the last two decades, a wide range of communication technologies have been designed or adapted for Internet of Things (IoT) applications. In this space, devices (e.g., sensors or actuators) are typically inexpensive machines with significant resource constraints and relaxed bandwidth requirements [1].

Most IoT technologies rely on some form of terrestrial infrastructure, with a coverage range that may span from a few meters up to several kilometers, depending on the technology [2]. However, when such infrastructure is not available, satellite communication technologies provide an alternative connectivity solution for IoT devices. In fact, satellite communication technologies have recently attracted the attention of the IoT community, since such technologies are experiencing a renaissance [3,4,5,6].

New satellite deployments are planned in the near future, while the number of satellite IoT connections is expected to grow by a factor of ~4 up to 2025 [6]. While early satellite systems made use of Geostationary Earth Orbit (GEO), most current and future deployments aim to leverage the lower altitude of Low Earth Orbit (LEO) systems, which offer lower cost, higher bandwidth, lower latency, and lower power consumption. Examples of current main LEO satellite systems include Iridium, Orbcomm, Globalstar, Argos, or Starlink [3].

A fundamental aspect of an IoT technology is its energy performance. This is especially critical for satellite IoT devices, which are typically not powered by the electrical grid. However, to the best of our knowledge, no previous work has focused on the energy performance of a satellite IoT technology. In this paper, we investigate the energy performance of Iridium satellite technology for IoT devices. We derive a current consumption model from measurements carried out on a real Iridium hardware module. We provide the average current consumption, theoretical device lifetime, and energy cost of data delivery results as a function of different communication protocol and link parameters. Our study shows that an Iridium-enabled IoT device, running on a 2400 mAh battery and sending a 100-byte message every 100 min, may achieve a theoretical lifetime of 0.95 years, whereas the maximum achievable lifetime for the same device (i.e., the asymptotic theoretical device lifetime as the message sending period increases) is 3.55 years. However, the device energy performance decreases significantly as the message rate increases compared to long-range IoT technologies such as LoRaWAN and Sigfox.

The remainder of the paper is organized as follows. Section 2 reviews the related work. Section 3 overviews the Iridium satellite system, along with the communications system used by Iridium IoT devices. Section 4 provides a current consumption model of Iridium IoT satellite technology. In Section 5, we use the model derived in the previous section to evaluate the energy performance of an Iridium IoT device. Section 6 compares the energy performance of Iridium with that of LoRaWAN and Sigfox. Section 7 concludes the paper with the main remarks from this work.

## 2. Related Work

This section reviews existing work that is related to the present paper. This section is divided in three parts, focusing on the following three areas, respectively: (i) satellite IoT from a broad perspective [3,4,5,6,7], (ii) Iridium satellite technology [8,9,10,11,12,13,14,15,16], and (iii) energy performance of IoT technologies [17,18,19,20,21,22].

### 2.1. Satellite IoT

A few published works have surveyed satellite IoT systems [3,4,5,6,7]. Centenaro et al. provided a comprehensive survey of the state-of-the-art in the area of satellite IoT, which focuses on current solutions, research, and industrial/commercial initiatives, and future research opportunities, among others [6]. De Sanctis et al. explored the use cases for satellite IoT, and pointed out open issues, especially in terms of connectivity and interoperability between sensors, satellites, and the Internet [7]. The authors created a taxonomy of satellite IoT systems, whereby end devices (e.g., sensors) use satellite connectivity directly or indirectly (in the latter, a terrestrial gateway covering a sensor network uses a satellite link for Internet connectivity). Fraire et al. analyzed the characteristics and open issues of direct-to-satellite systems [3]. Qu et al. focused on the benefits of LEO satellite systems for IoT and provided a holistic LEO satellite architecture overview [5]. Ferrer et al. reviewed the panoply of medium access control (MAC) protocols for satellite IoT systems, with a focus on low-cost nanosatellite systems [4].

### 2.2. Iridium Satellite Technology

While a full Iridium system description or specification is not publicly available, several research works have overviewed the Iridium technology [8,9], evaluated its performance [9,10], or used it for various kinds of applications [11,12,13,14,15,16].

Maine et al. presented the main concepts, architecture, and services of the Iridium system [8]. Pratt et al. describe the system fundamentals in more detail, and provide a network performance analysis in terms of communication latency [9]. McMahon et al. performed an experimental latency characterization of Iridium [10].

Iridium has been used as the communication means in many scenarios for both voice and data communications [9,12]. Among the latter, Iridium has enabled many IoT use cases in a diversity of remote scenarios including polar, marine, desert, forest, and UAV-based, among others [11,12,13,14,15]. Iridium has also been exploited for positioning applications [16].

### 2.3. Energy Performance of IoT Technologies

Since many IoT devices are powered by limited energy sources, energy performance is a crucial aspect of an IoT technology. Many studies have focused on modeling, evaluating, and/or improving the energy performance of a wide range of IoT technologies such as IEEE 802.15.4 and ZigBee [17], Bluetooth Low Energy (BLE) [18,19], LoRaWAN [20], Sigfox [21], or NB-IoT [22], among others. However, to our best knowledge, no work in the literature has evaluated the energy performance of a satellite technology for IoT.

## 3. Iridium Overview

The Iridium satellite system was originally conceived to comprise 77 satellites, which inspired the name for the system, since the atomic number of the iridium chemical element is 77. The Iridium system finally consisted of 66 satellites. 

While most previously existing satellite communication systems used GEO satellites, Iridium was designed based on LEO satellites. This feature was a consequence of the need to offer worldwide voice and data communications coverage for lightweight handheld devices. For such use cases, LEO satellites require lower transmission power and offer lower propagation delay. The altitude of Iridium satellites was around 780 kms. The system became commercially available in 1998. In 2019, the launch of a new generation of Iridium satellites, called Iridium Next, was completed. There are currently 75 Iridium Next satellites in orbit, comprising 66 operational satellites, and nine on-orbit spare satellites. Note that Iridium Next satellites are fully compatible with the initial Iridium system. The altitude of 70 such satellites is 625 kms, whereas the altitude of the remaining five satellites is 720 kms [16].

**Figure 1:**
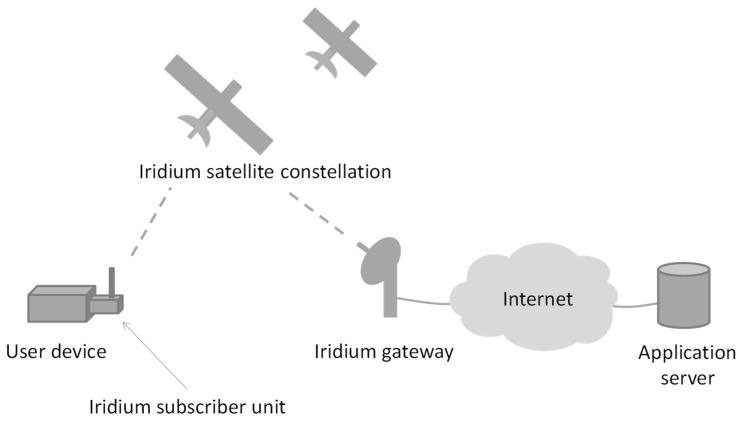
Iridium satellite system architecture.

Figure 1 illustrates the architecture of the Iridium satellite system. An Iridium subscriber unit (i.e., the Iridium modem of the user device) communicates via a radio link with the Iridium satellite constellation. Data from the user device may be routed through several satellites until they are transmitted in the downlink to an Iridium terrestrial gateway or to another user. An Iridium gateway is connected to the Internet, where data can reach an application server.

Each satellite supports three antenna arrays supporting 16 beams each, providing a total of 48 spot beams on the Earth’s surface, effectively defining 48 covered cells. Note that Iridium satellites are not geostationary (i.e., they do not remain in the same location). Iridium satellites travel at 26,804 km/h, resulting in a complete orbital period of 100.13 min [9]. As a consequence, from a given ground location, a satellite is visible for seven minutes. When a satellite becomes unreachable for a user, an attempt is performed to hand communication to another satellite. In order to enable intersatellite links, satellites are equipped with additional antennas.

Iridium uses a portion of L-band spectrum, ranging from 1616 MHz to 1626.5 MHz, for communication. The frequency band between 1616 MHz and 1626 MHz is used to support duplex user communication. The band between 1626 MHz and 1626.5 MHz is used for simplex downlink signaling. The duplex band is divided by means of frequency division multiple access (FDMA) into 240 frequency channels of 41.67 kHz bandwidth each, with an operation bandwidth of 31.5 kHz and guard bandwidth of 10.17 kHz. A frequency channel may in turn be shared among different users by means of a time division multiple access (TDMA) scheme. A TDMA frame is defined with a total duration of 90 ms, which comprises an initial signaling simplex header, four uplink timeslots, and four downlink timeslots. The simplex header has a duration of 20.32 ms, whereas each uplink or downlink timeslot has a duration of 8.28 ms. The remaining time up to the total duration of 90 ms corresponds to guard times before and after the simplex header and each timeslot [9]. A frame supports duplex communication for four simultaneous users by using time division duplex (TDD). Each timeslot contains a burst composed of three parts: an unmodulated tone, a unique word, and the data field. The unique word is transmitted by using binary phase shift keying (BPSK) modulation, whereas the data field is transmitted by means of quadrature phase shift keying (QPSK) modulation [16]. One frame carries a total of 4500 bits, leading to a bit rate of 50 kbit/s. A user duplex timeslot carries a total of 414 bits.

Iridium offers different types of communication services including dial-up, direct Internet access, paging, router-based unstructured digital inter-working connectivity solution (RUDICS), and short burst data (SBD). For IoT purposes, that is, infrequent transmission of short payloads (e.g., corresponding to sensor readings) with relaxed latency requirements (e.g., in the order of tens of seconds), SBD is the most suitable service. 

SBD is a packet-switched-oriented service that uses forward error correction (FEC) encoding, along with a selective automatic repeat request (ARQ) mechanism. In SBD, the user duplex timeslot comprises 160 SBD information bits, along with 20 SBD header bits, and 234 bits that correspond to other overheads. The 160 SBD information bits are protected by BCH(31,20) FEC encoding [23]. The theoretical user data capacity of a SBD channel (i.e., 160 user data bits are carried every 90 ms) is 1.78 kbit/s. However, the actual user capacity defined for a SBD channel is 125 byte/s (i.e., 1 kbit/s). The maximum size of a message sent by a user (e.g., a sensor device equipped with Iridium satellite connectivity) is 1960 bytes. However, commercial devices may support a smaller maximum message size (e.g., 340 bytes in the RockBLOCK Mk2 model [24]). In fact, IoT applications typically handle small user payloads.

An Iridium IoT device can send and receive data by means of the aforementioned SBD duplex timeslots. When a message is sent to the device, it is stored in a queue at the gateway. If the gateway receives a message from the Iridium IoT device, the first message in the queue is then sent to the device. Therefore, downlink data transmission requires a previous uplink transmission performed by the Iridium IoT device.

## 4. Current Consumption Model for Iridium Satellite IoT

This section presents a current consumption model of a device that periodically transmits a message (e.g., a sensor reading) with payload size *L*, once every time interval, *T*, by means of the Iridium SBD service. We assume that the device is by default in sleep mode in order to save energy, except when it executes the operations required for message transmission. Based on the current consumption model, the section also shows how the device battery lifetime and energy efficiency of data delivery can be calculated.

In order to provide a realistic model, we performed current consumption measurements on a real device hardware platform, called RockBLOCK Mk2 (Rock Seven Mobile Services, Whiteley, UK). This platform uses the Iridium 9602 module, and supports the Iridium SBD service, with a maximum transmitted message size of 340 bytes. The transmit power used by the device is 32 dBm. Note that the device includes a supercapacitor, which acts as an energy reservoir and allows to buffer the highly pulsed nature of the device internal circuits.

**Figure 2:**
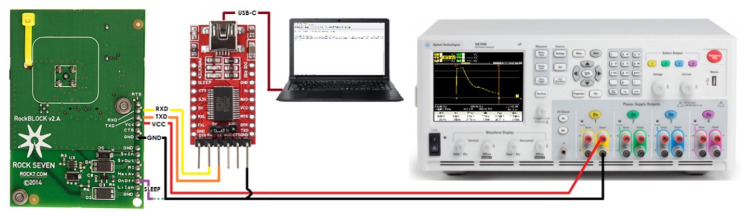
Experimental setup for measuring the current consumption of the RockBLOCK Mk2 Iridium IoT device.

Figure 2 illustrates the experimental setup used for the measurements. The RockBLOCK device (left) was controlled via AT commands from a computer, while an Agilent Technologies N6705A power analyzer (right) was used to measure the current consumption of the device.

**Figure 3:**
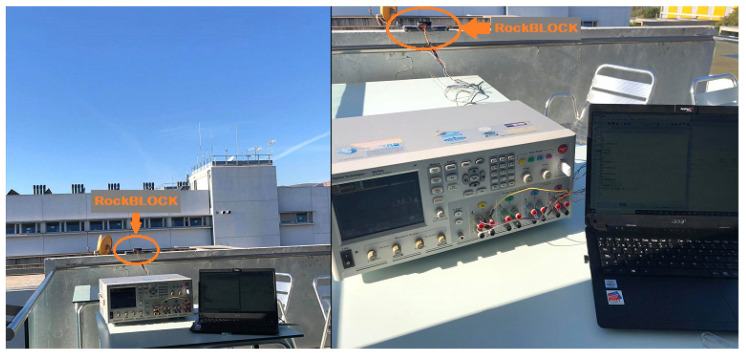
Outdoor scenario used for the current consumption measurements of the RockBLOCK Mk2 Iridium IoT device.

Measurements were performed in an outdoor scenario, in line-of-sight conditions (i.e., without obstacles between the RockBLOCK device and the Iridium satellites). Figure 3 shows the location of the RockBLOCK device, and the rest of the experimental setup, in the mentioned outdoor scenario. 

**Figure 4:**
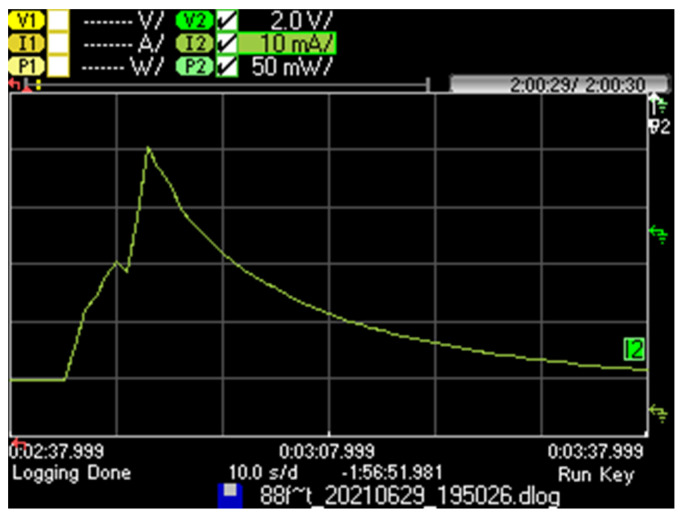
Current consumption pattern of a RockBLOCK Mk2 device that is initially in sleep state, performs a successful message transmission, and returns to sleep state.

We next provide the current consumption pattern and main characteristics for the considered Iridium IoT device for a successful message transmission, for *L* = 1 byte (see Figure 4). The device is initially in a sleep state, then it becomes active to perform a successful message transmission, and finally, it returns to the sleep state.

Remarkably, while the device is active, it spends an average of 6.0 s involved in tasks that do not correspond to payload transmission such as device processing, protocol overhead, etc. This time is significant, even when compared with the nominal data transmission time of up to 2.72 s for the maximum payload size allowed in SBD (i.e., 340 bytes). Recall that, as mentioned in Section 3, despite the theoretical user capacity of 1.78 kbit/s, Iridium SBD offers an actual user capacity of 1 kbit/s. Payload transmission time is determined as the payload size in bits divided by the user capacity of 1 kbit/s. On the other hand, the device consumes a greater amount of current after transmission has finished, during which the device returns to the sleep state and its current consumption decreases slowly. Such a slow decrease is due to the supercapacitor included in the device. The average current consumption of the considered successful message transmission (computed since the device receives an AT command to start message transmission, until 3 min later), denoted as *I_OK_*, is 7.66 mA. The current consumption of the device when it returns to sleep mode tends asymptotically to be 77.0 μA, after more than 24 h in the absence of communication activity.

**Figure 5:**
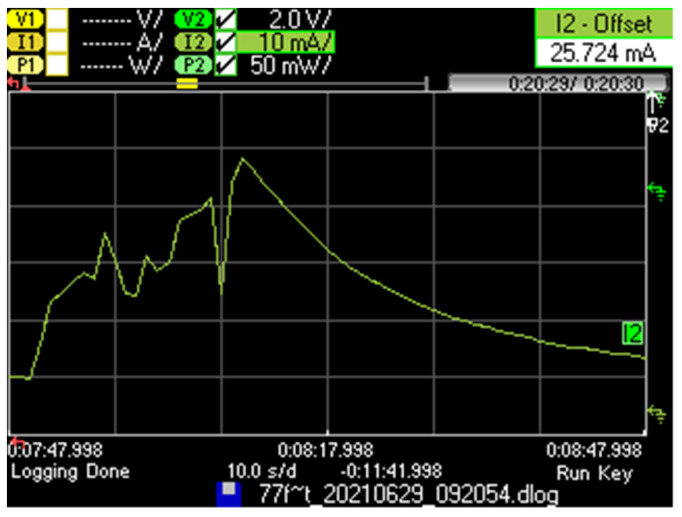
Current consumption pattern of a RockBLOCK Mk2 device that is initially in sleep state, performs an unsuccessful message transmission attempt, and returns to sleep state.

We next focused on the current consumption behavior of the Iridium IoT device when it performs a message transmission in apparently good visibility conditions, but the message cannot be successfully delivered, for *L* = 1 byte (Figure 5). In line-of-sight conditions, such an event may mainly occur due to an intersatellite handover or due to contention for communication resources. We observe that, in this case, the average current consumption of the device increased; we speculate that the device performs lower layer retransmission attempts, and after unsuccessful message delivery, the message transmission is considered failed, and the device returns to sleep mode. However, additional upper-layer retransmissions of the same message can be triggered subsequently by the application.

Based on our measurements, we found that the average time since the start of the operation until the instant in which the device returns to sleep mode, when message delivery is unsuccessful, is 20.0 s. This duration, which is greater than the one in a successful message transmission, is consistent with our speculation that lower layer retries may have been performed. 

The average current consumption corresponding to the considered unsuccessful message transmission, since the device receives the command to start message transmission until 3 min later, denoted *I_KO_*, is 10.1 mA.

**Figure 6:**
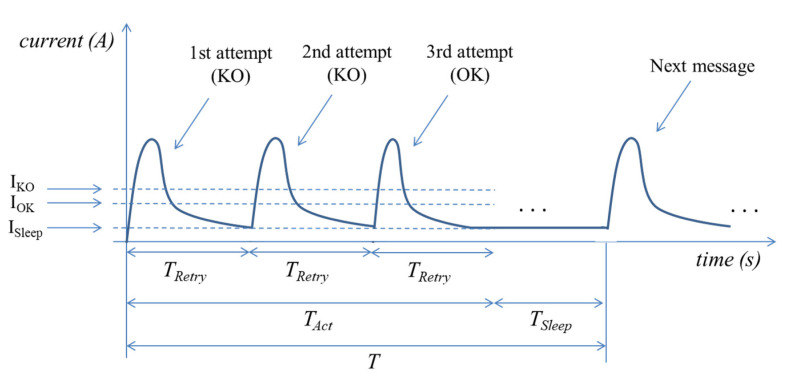
Device current consumption over time, where three transmission attempts are needed for successful delivery of a message. The main current and time parameters of the model are indicated in the figure.

We next calculated the average current consumption of the considered Iridium device, denoted *I_Avg_*, taking into account that unsuccessful message transmission may occur, with probability *p*, and assuming that an application-layer mechanism triggers a message retransmission after *T_Retry_* if the last message transmission attempt has failed. We also assume that successive retries of a failed transmission attempt are mutually independent. Figure 6 illustrates the device current consumption in an example where three transmission attempts are needed for successful delivery of a message.

Let *T_Act_* denote the duration of the interval since the Iridium device starts the process to perform a message transmission, until the message is successfully delivered. Note that, after an unsuccessful message transmission, there will be an application-layer-triggered message retransmission. The time between the start of two consecutive message transmission attempts is equal to *T_Retry_* (see Figure 6).

Let *T_Sleep_* denote the time since successful message transmission until the start of the first transmission attempt of the next message. We assume *T_Act_* < *T*, which will hold in a practical scenario. Therefore, *T_Sleep_* + *T_Act_* = *T*.

Let *I_Act_* and *I_Sleep_* indicate the average current consumption of the device during *T_Act_* and *T_Sleep_*, respectively.

Based on the defined variables, the average current consumption of the device, *I_avg_*, can be calculated as follows:

(1) $$I_{Avg}=\frac{E\left[I_{Act}\right]\cdot E\left[T_{Act}\right]+E\left[I_{Sleep}\right]\cdot E\left[T_{Sleep}\right]}{T}$$

where *E[·]* indicates the expected value of the corresponding variables. 

We started to calculate the terms in (1) by first determining *E[I_Act_]*. For the sake of analytical simplicity, we assumed that *T_Act_* comprises an integer number of intervals of duration *T_Retry_* including the interval where the successful message transmission occurs (i.e., the last interval, see Figure 6). Let *I_OK_* and *I_KO_* denote the average current consumption of an interval of duration *T_Retry_*, where successful and unsuccessful message transmission have occurred, respectively. Let *n* indicate the number of intervals of duration *T_Retry_* that may occur within *T*. 

Based on the introduced variables, *E[I_Act_]* can be computed as shown next:

(2) $$E\left[I_{Act}\right]=I_{OK}\cdot \left(1-P\right)+\frac{I_{OK}+I_{KO}}{2}\cdot P\cdot \left(1-P\right)+\frac{I_{OK}+2\cdot I_{KO}}{3}\cdot P^{2}\;\cdot \left(1-P\right)\;+\cdot \;\cdot \;\cdot +\frac{I_{OK}+\left(n-1\right)\cdot I_{KO}}{n}\cdot P^{n-1}\;\cdot \left(1-P\right)$$

The above equation can be expressed as follows:

(3) $$E\left[I_{Act}\right]=\overset{n}{\underset{i=1}{\sum }}\frac{I_{OK}+\left(i-1\right)\cdot I_{KO}}{i}\cdot P^{i-1}\;\cdot \left(1-P\right)$$

We next determined *E[T_Act_]*, under the considered assumptions. *E[T_Act_]* can be determined as follows:

(4) $$E\left[T_{Act}\right]=T_{Retry}\cdot \left(1-P\right)+2\cdot T_{Retry}\cdot P\cdot \left(1-P\right)+3\cdot T_{Retry}\cdot P^{2}\;\cdot \left(1-P\right)+\;\cdot \;\cdot \;\cdot +n\cdot T_{Retry}\cdot P^{n-1}\;\cdot \left(1-P\right)$$

The above equation can be expressed as shown next:

(5) $$E\left[T_{Act}\right]=T_{Retry}\cdot \left(1-P\right)\overset{n}{\underset{i=1}{\sum }}i\cdot P^{i-1}$$

The next term from (1) that we determined is *E[T_Sleep_]*, which can be easily found as follows:

(6) $$E\left[T_{Sleep}\right]=T-E\left[T_{Act}\right]$$

Finally, *E[I_Sleep_]* can be determined empirically. This value decreases with *T*, and it tends asymptotically to 80.7 μA.

Therefore, *I_avg_* can be calculated based on the presented Equations ((1)–(6)) and values. 

Assuming that the Iridium IoT device runs on a battery of capacity *C_batt_*, the theoretical lifetime of the device can be calculated as follows:

(7) $$Lifetime=\frac{C_{batt}}{I_{Avg}}$$

Note that the theoretical device lifetime calculated as per (7) assumes a battery of ideal characteristics. A real battery will typically exhibit non-ideal features (e.g., a certain degree of self-discharge) that will lead to a lower actual device lifetime. Therefore, the theoretical device lifetime must be considered as an upper bound of the actual device lifetime. 

The last performance metric th we considered was the energy efficiency of data transmission when using the Iridium device. This metric can be calculated in terms of the energy cost per delivered user data bit, *EC_db_*, as shown next, where *V* indicates the battery voltage:

(8) $$EC_{db}=\frac{I_{Avg}\cdot V\cdot T}{L}$$

## 5. Evaluation

On the basis of the models provided in the previous section, this section evaluates the average current consumption and the lifetime of the considered Iridium IoT device as well as the corresponding energy cost per delivered bit. The section is organized into three subsections, which focus on each of the aforementioned performance parameters, respectively. 

We performed the evaluation for a wide range of *T* values. We also considered different values for *p*, which allowed us to capture ideal and non-ideal conditions (note that, in absence of application-triggered retries, we experimentally observed a value of *p* ≈ 0.1). We assumed *T_Retry_* = 3 min.

### 5.1. Device Current Consumption

**Figure 7:**
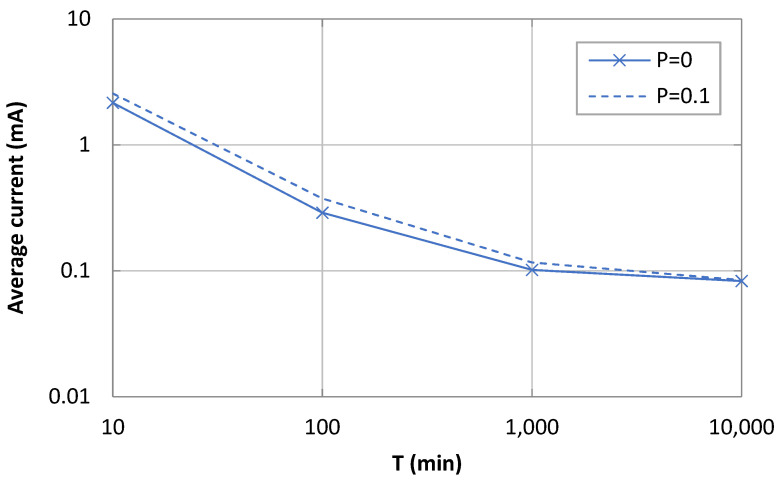
Average current consumption as a function of *T*.

Figure 7 illustrates the average current consumption of the device as a function of *T*, for different *p* values, based on the model in Section 4. As expected, the average current consumption decreased with *T* as the influence of sleep intervals increased, and the average current consumption tends asymptotically to sleep state current consumption.

The average current consumption increased with *p*, since the number of application-layer retries also increases. However, impact of *p* on the average current consumption varies depending on the value of *T*. For *p* = 0.1, and relatively low values of *T* (e.g., *T* = 10 min), the average current consumption increases by a factor of ~20%. As *T* increases (e.g., for *T* = 100 min), such a factor reaches ~31%, since intervals where the device slowly return to sleep become more significant (with greater duration and lower current consumption), and retries have a relatively greater impact than for smaller values of *T*. As *T* increases further, the active intervals related with message transmission become less relevant compared with very long sleep intervals, rendering the active intervals asymptotically negligible. 

We also analyzed the impact of the payload size on the average current consumption. Note that payload transmission time contributes to both *I_OK_* and *I_KO_*, therefore, it also contributes to *E[I_Act_]*. As payload size increases, *E[I_Act_]* also increases, which in turn increases the average current consumption. While such an impact decreases with *T*, we found that payload size actually had a low influence on the average current consumption for the whole range of considered *T* values (of 6.5% for *T* = 10 min, and below 1% for the rest of the considered *T* values). For the sake of illustration clarity, Figure 6 only shows the results for a 1-byte message payload size, since curves for the rest of the considered payload sizes (*L* ≤ 300 bytes) show negligible differences. 

**Figure 8:**
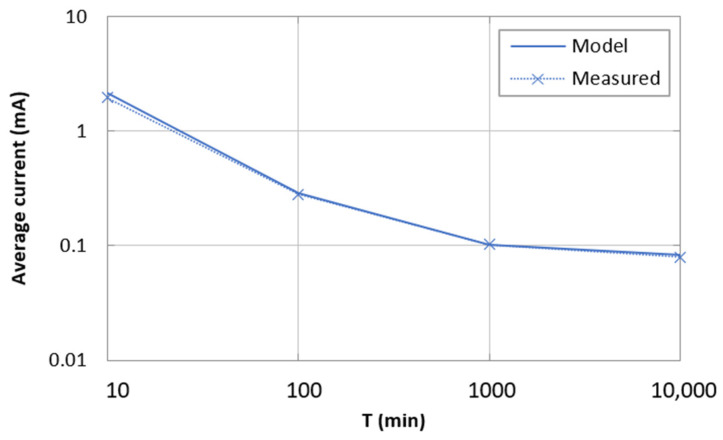
Average current consumption as a function of T: model (*p* = 0) vs. measured results.

Figure 8 also depicts the average current consumption of the device as a function of T. In this case, the figure compares the results provided by the model with results obtained by measuring the average current consumption over intervals of a duration one order of magnitude greater than each corresponding value of T (except for T = 10,000 min, where the duration was equal to T). In the measurements, all transmissions were successful, therefore we show the model results for *p* = 0. As shown in Figure 8, the model generally predicts current consumption with high accuracy, especially for T ≥ 100 min. For T = 10 min, the difference between the model and the measured results is greater (equal to 10%), since the transmission part, which is prone to variability depending on communication conditions, becomes more relevant.

### 5.2. Device Lifetime

We next evaluate the theoretical lifetime of an Iridium device, based on (7), and considering a battery capacity of 2400 mAh, as a function of *T* and *p*.

**Figure 9:**
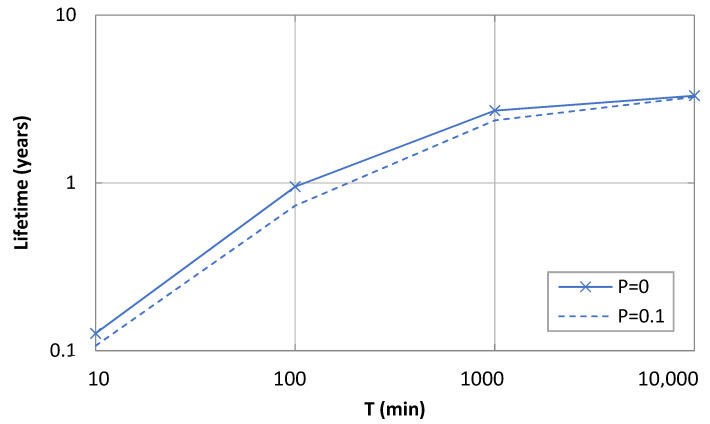
Lifetime of a 2400 mAh-battery-enabled device as a function of *T*.

As shown in Figure 9, lifetime increases asymptotically with *T*. The maximum lifetime was 3.39 years. Remarkably, a lifetime of one year could only be achieved for values of *T* greater than 100 min. As *T* decreased below 100 min, device lifetime decreased abruptly. For example, the device lifetime for *T* = 10 min was only 43.8 days.

As expected, device lifetime also decreases with *p* due to the greater current consumption caused by retries. The impact of a non-zero *p* on asymptotic device lifetime, for practical link qualities, is negligible. 

### 5.3. Energy Cost per Delivered Bit

**Figure 10:**
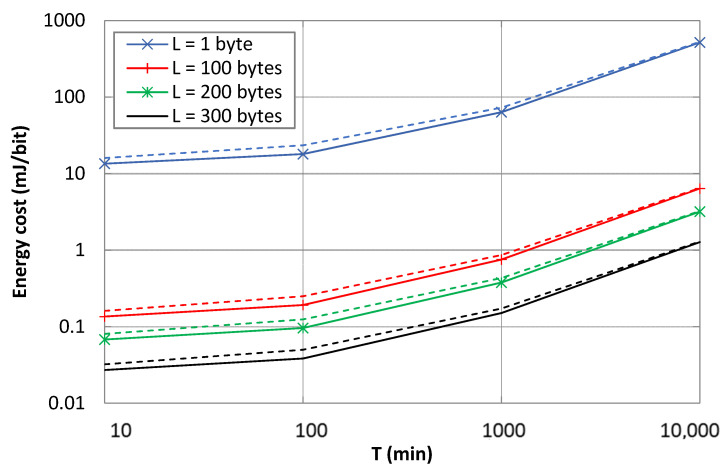
Energy cost vs. T, for several message payload sizes and *p* values. Dashed curves represent the energy cost values for *p* = 0.1 for the payload sizes illustrated with the same color.

Figure 10 illustrates the energy cost per delivered bit of user data, as a function of *T*, *L*, and *p*, based on (8). This performance parameter increases with *T* due to the energy consumed during sleep intervals. On the other hand, increasing the user data payload size amortizes the energy required to deliver a user data bit. The latter may be reduced by up to between two and three orders of magnitude.

Message delivery errors increase the energy cost per delivered bit due to the additional cost of retransmissions as well as the greater energy consumption of a failed message transmission attempt.

## 6. Comparison with Other IoT Technologies

In order to assess the energy performance results obtained in the previous section from a broader perspective, this section compares the energy performance of Iridium satellite IoT technology with that of prominent long-range terrestrial IoT technologies such as LoRaWAN or Sigfox. The two latter technologies offer a communication link range typically 1–2 orders of magnitude lower than the Iridium one. While energy performance of a given technology depends on how the technology is configured, and on how it is implemented on real hardware, this section aims to explore the energy performance of the different communication technologies on representative examples using reasonably common settings.

**Figure 11:**
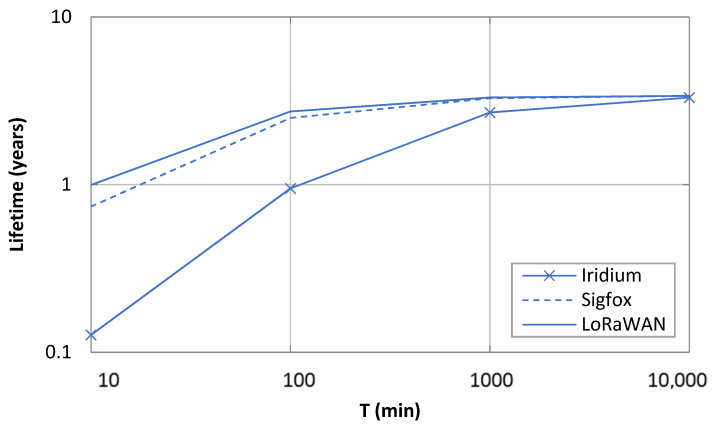
Lifetime of a device running on a 2400 mAh battery for Iridium, Sigfox. and LoRaWAN, as a function of *T* and for *p* = 0.

Figure 11 depicts the lifetime of a 2400 mAh-battery-operated device sending a message (carrying a 12-byte payload) for different values of *T* and for *p* = 0, for Iridium, Sigfox, and LoRaWAN. In all cases, an ideal line-of-sight channel has been assumed. In addition, a single user was assumed (i.e., there was no interference from other users). For Sigfox and LoRaWAN, the calculations were performed by using published energy models and device settings for operation in the 868 MHz band [20,21]. More specifically, for the Sigfox evaluation, radio configuration (RC) number 1 (i.e., RC1) and unidirectional data message transmission were assumed [21]. In RC1, the uplink and downlink bit rate for RC1 was 100 bit/s. For the LoRaWAN evaluation, the sender device belonging to Class A, and data rate (DR) 0, which corresponds to spreading factor (SF) 12 and a bit rate of 250 bit/s, has been used [20]. Under the considered conditions, and due to spectrum access regulations, both LoRaWAN and Sigfox need to enforce a duty cycle below 1%. Note that Iridium satellite IoT technology is also assumed to be duty cycled in this study, since for feasibility and for a practical battery lifetime, the IoT device must return to sleep mode after message transmission.

For a fair comparison of the asymptotic device lifetime (which is independent of communication activity), the same battery capacity and asymptotic sleep current have been assumed for all three considered technologies. Note that while the transmission power used by the Iridium device was 32 dBm, the transmit power considered in the Sigfox and LoRaWAN energy models used were 14 dBm and 11 dBm, respectively. These transmit power settings stem from the need to comply with European regulations on the frequency bands used by Sigfox and LoRaWAN (which limit transmit power to 14 dBm). 

As shown in Figure 11, as *T* increases, device battery lifetime becomes less dependent on the communication technology, since sleep intervals become dominant. However, as *T* decreases, the device lifetime for Iridium becomes significantly lower than that achieved for LoRaWAN and Sigfox (e.g., by factors of 7.8 and 5.8 for *T* = 10 min, respectively). Such a difference cannot only be attributed to the transmission power of the Iridium device, which leads to a current consumption of up to 45.8 mA (Figure 4), since the current consumption in transmit state for the LoRaWAN and Sigfox devices is 82.8 mA and 27.5 mA, in their respective energy models. There are two main contributors to Iridium device energy consumption underperformance: (i) the current consumption penalty introduced by its supercapacitor, especially when the device returns to sleep mode after communication activity, and (ii) the large processing and communication overhead, which corresponds to 6.0 s (for a nominal transmission time of 2.72 s for a 340-byte user payload) during which the device consumes a significant amount of current.

## 7. Conclusions

To our best knowledge, in this work, we provided the first energy performance study of a satellite IoT technology. We first performed a current consumption characterization of an Iridium device, based on real hardware. On that basis, we derived a current consumption model and evaluated the energy performance of an Iridium device in terms of average current consumption, device lifetime, and energy cost of data delivery. We also compared the energy performance of Iridium technology for IoT use cases with that of well-established long range IoT technologies such as LoRaWAN and Sigfox.

Energy-wise, Iridium is a suitable technology for relatively infrequent message transmission. The asymptotic lifetime of the battery-operated Iridium IoT device considered, for a battery capacity of 2400 mAh, is 3.55 years. For very low message rates (e.g., once per day), it performed similarly to LoRaWAN or Sigfox. However, Iridium energy underperformance increases with message rate, compared to the latter technologies. However, Iridium still offers nearly a one-year lifetime for a rate of one message every 100 min.

This work focused on the use case of a battery-operated IoT device using Iridium to send messages periodically such as a sensor. However, the work also allows us to conclude that a battery-operated actuator using Iridium might only be feasible for latency-tolerant applications. Since delivering a message (e.g., a command) to such an Iridium device requires the device itself to previously send a data message, and the time between uplink message transmissions needs to be relatively long for a practical battery lifetime, sending a command to an actuator may involve a significant delay (e.g., in the order of hours, or even greater).

## Data Availability

Not applicable.
